# Conscious and unconscious context-specific cognitive control

**DOI:** 10.3389/fpsyg.2014.00539

**Published:** 2014-06-04

**Authors:** Nathalie Schouppe, Evelien de Ferrerre, Filip Van Opstal, Senne Braem, Wim Notebaert

**Affiliations:** Department of Experimental Psychology, Ghent UniversityGhent, Belgium

**Keywords:** masked priming, consciousness, cognitive control, CSPC effect, context

## Abstract

A key feature of the human cognitive system is its ability to deal with an ever-changing environment. One prototypical example is the observation that we adjust our information processing depending on the conflict-likelihood of a context (context-specific proportion congruency effect, CSPC, Crump etal., 2006). Recently, empirical studies started to question the role of consciousness in these strategic adaptation processes (for reviews, see Desender and Van den Bussche, 2012; Kunde etal., 2012). However, these studies have not yielded unequivocal results (e.g., Kunde, 2003; Heinemann etal., 2009; Van Gaal etal., 2010a; Desender etal., 2013; Reuss etal., 2014). In the present study, we aim at replicating the experiment of Heinemann etal. (2009) in which the proportion of congruent and incongruent trials between different contexts was varied in a masked priming task. Their results showed a reduction of the congruency effect for the context with more incongruent trials. However, this CSPC effect was only observed when the prime–target conflict was conscious, rather than unconscious, suggesting that context-specific control operates within the boundaries of awareness. Our replication attempt however contrasts these findings. In the first experiment we found no evidence for a CSPC effect in reaction times (RTs), neither in the conscious nor in the unconscious condition. The error rate analysis did show a CSPC effect, albeit not one modulated by consciousness. In the second experiment we found an overall CSPC effect in RTs, independent of consciousness. The error rates did not display a CSPC pattern. These mixed results seem to nuance the findings of Heinemann etal. (2009) and highlight the need for replication studies in psychology research.

## INTRODUCTION

The role of consciousness is a hotly debated topic in cognitive psychology. While it is now firmly established that unconscious stimuli can be processed up to a semantic level (Greenwald et al., 1996; Dehaene et al., 1998; Van den Bussche et al., 2009), it remains a matter of debate whether unconscious stimuli can also influence other higher order cognitive functions, such as cognitive control and decision making. Some researchers have claimed that this cannot be the case, stating that “it should be impossible for an unconscious stimulus to modify processing on a trial-by-trial basis through top-down control” (Dehaene and Naccache, 2001, p. 21; for reviews see Hommel, 2007; Kunde et al., 2012; Van Gaal et al., 2012). Yet, several empirical findings challenge this idea, showing how unconscious stimuli can trigger inhibitory control processes (Van Gaal et al., 2009, 2010b) and lead to post-error (Hester et al., 2005; Cohen et al., 2009) and post-conflict (Van Gaal et al., 2010a; Desender et al., 2013) behavioral adjustments. The evidence is however far from unequivocal, highlighting the need to replicate some of the initial studies.

In what follows, we will specifically focus on adaptations to (unconscious) conflict as an instance of cognitive control. We will review studies questioning whether these conflict-driven adjustments can occur with or without awareness. However, these studies on unconscious conflict adaptation all have one potential drawback, namely that the effect under investigation can also be explained by simple episodic memory learning processes (Hommel, 1998; Schmidt and Besner, 2008; Schmidt, 2013), without cognitive control meddling in (but see Duthoo and Notebaert, 2012). In other words, we can question whether the results reflect unconscious implementation of control processes or episodic learning processes. One study on cognitive control and consciousness partly addressed this issue such that one particular explanation in terms of event learning could be ruled out (Heinemann et al., 2009; see also Crump and Milliken, 2009). This study of Heinemann et al. (2009) will be the focus of the present replication study.

### CONFLICT PROCESSING AND COGNITIVE CONTROL

In the laboratory, cognitive control processes are typically investigated by means of interference tasks. These tasks all have in common that they require responding to a task-relevant stimulus, while simultaneously ignoring irrelevant, competing information. In a number priming task for instance, participants are asked to respond to a target number (e.g., indicate whether the number is smaller or larger than 5) while ignoring a prime number, which is presented briefly before the target. The design of this task implies a distinction between congruent and incongruent trials. The former trial type consists of trials where both prime and target activate the same response (e.g., “2” as the prime and “4” as the target), whereas the latter is composed of those trials where prime and target activate a different response (e.g., “2” as the prime and “8” as the target). Not surprisingly, incongruent trials lead to slower and more error-prone responses compared to congruent trials (i.e., congruency effect).

Importantly however, it has been shown that participants can adapt to the conflict elicited by incongruent trials. For instance, it is a well-known observation that the congruency effect can be modulated on a trial-by-trial basis, with a smaller congruency effect following an incongruent trial, relative to a previous congruent trial (i.e., congruency sequence effect, for a review, see Egner, 2007). Also, when varying the proportion of congruent and incongruent trials in different contexts, it has been shown that the congruency effect is reduced in a context signaling a high degree of conflict (i.e., context-specific proportion congruency effect, CSPC effect, for a review, see Bugg and Crump, 2012). These effects have typically been explained as reflecting a strategic up-regulation of cognitive control after the occurrence of conflict (Botvinick et al., 2001), leading to better task performance after previous incongruent trials (i.e., congruency sequence effect) and in a context with a high proportion of incongruent trials (i.e., CSPC effect).

However, alternative explanations for these effects in terms of lower level episodic learning processes have been put forward (Hommel, 1998; Mayr et al., 2003; Schmidt, 2013). For instance, Hommel (1998) explained the congruency sequence effect by claiming that stimulus and response features of a particular trial are bound in an episodic memory code, leading to faster responses on the next trial when all features completely repeat or alternate, than when there is only a partial repetition/alternation of features. Similarly, it has been suggested that in contexts with a high proportion of (in)congruent trials, the frequent occurrence of specific stimulus–response–context combinations leads to increased event binding and hence modulations of the congruency effect. Several studies however showed that the congruency sequence effect persists after excluding all feature repetitions (Notebaert and Verguts, 2007; Duthoo and Notebaert, 2012), and that the CSPC effect is still present on trials for which the specific prime–target–context combinations are equally frequent in both contexts (Crump and Milliken, 2009).

Unfortunately, controlling for these alternative accounts is not yet common practice in research on the consciousness-control link. This puts into question whether some studies truly investigate the role of consciousness in cognitive control, since an interpretation in terms of event learning is often not excluded. For the present research, it is especially important to rule out these episodic learning accounts because most consciousness theories agree on the idea that low-level bottom-up learning mechanisms as described by Hommel (1998) and Schmidt (2013) do not require consciousness. Therefore, by controlling for these bottom-up explanations, we can investigate a “purified” indexation of top-down cognitive control. This is crucial, because, while agreeing on the idea that the abovementioned episodic learning effects can work unconsciously, consciousness theories differ in their predictions for cognitive control. The global neuronal workspace (Dehaene and Naccache, 2001) for example would argue that consciousness is a prerequisite for frontal lobe processes, such as top-down control. Other theories (e.g., Lamme and Roelfsema, 2000) refrain from tying consciousness to the frontal cortex (and hence to cognitive control).

### CONSCIOUSNESS AND COGNITIVE CONTROL

Until now, several studies have investigated whether participants can adjust their performance after unconscious conflict (for a review, see Desender and Van den Bussche, 2012), yet the findings are mixed. In the study of Kunde (2003), participants had to indicate the direction of a target arrow, which was preceded by a smaller prime arrow, that could either match (congruent trial) or mismatch (incongruent trial) with the direction of the target. Importantly, the prime arrow could fit into the contour of the target arrow such that it was rendered invisible when prime and target were presented in rapid succession (i.e., meta-contrast mask). Kunde (2003) observed a typical congruency sequence effect when the prime was consciously perceived. However, this effect disappeared when the prime was invisible, suggesting that cognitive control adjustments could only be implemented when the conflict was consciously visible. Ansorge et al. (2011) and Frings and Wentura (2008) came to the same conclusion using different conflict tasks and masking procedures. In these studies, the congruency sequence effect was also only evident when the primes were visible.

In contrast to these studies, Van Gaal et al. (2010a) could provide evidence for an unconsciously triggered congruency sequence effect. Their experiment was identical to Kunde (2003), except for two modifications. They shortened the intertrial-interval and removed the auditory signal that Kunde (2003) presented before each trial. Van Gaal et al. (2010a) showed that even after unconsciously perceived conflict, participants adjusted their performance, leading to a smaller congruency effect after unconscious incongruent trials compared to unconscious congruent trials. Desender et al. (2013) now also replicated this finding, thereby arguing that cognitive control does not require consciousness.

However, as argued above, the congruency sequence effect can be explained – albeit not exclusively – by other accounts, for which these studies did not control. Only one study on cognitive control and consciousness took this into consideration, providing a first attempt to differentiate between a cognitive control and event learning account. More specifically, the study of Heinemann et al. (2009) used a context-specific proportion congruency manipulation to investigate whether cognitive control operates within the boundaries of awareness. In their study, participants had to judge the magnitude of a target number that was preceded by a prime number that could either facilitate or interfere with the target response. This priming task was embedded in two contexts, with one context containing 80% congruent and 20% incongruent prime–target combinations (low-conflict context), and the other context consisting of 20% congruent and 80% incongruent prime–target combinations (high-conflict context). In the first experiment, the prime numbers were clearly visible to the participants, whereas in the second experiment the prime numbers were heavily masked, such that the conflict between prime and target responses was unconscious. Crucially, Heinemann et al. (2009) included test trials with equal frequencies in both contexts (see also Crump and Milliken, 2009). Heinemann et al. (2009) found a CSPC effect on the test trials, only in the conscious condition, and not in the unconscious condition.

The study of Heinemann et al. (2009) is thus especially important, since they did not find evidence for unconscious cognitive control, while excluding an alternative interpretation in terms of event learning processes. Moreover, given the mixed findings in the field, replication is crucial. Unfortunately, null findings are less likely to get published. This is also highlighted by Kunde et al. (2012, p. 15), stating: “research on the consciousness-control link is perhaps particularly susceptible to publication bias. Positive evidence for control without awareness is exciting and may make it easier to be published in prestigious journals (or to be published at all), while negative evidence resides in less prestigious journals (or may not be published).” We therefore aimed at replicating the results of Heinemann et al. (2009), and predicted the absence of a CSPC effect in the unconscious condition. As will be clear from the description below, the first experiment is not an exact replication, differing in some small aspects from the original study. Therefore, we also conducted a second experiment, in which we more accurately replicated the procedures as described by Heinemann et al. (2009).

## EXPERIMENT 1

### PARTICIPANTS

Forty students (*M*: 22.5 years of age, SD: 2.3; 26 female) from Ghent University participated in the study. They had normal or corrected-to-normal vision. All participants provided written informed consent and received 24 euro in return for their participation.

### STIMULI AND MATERIAL

Participants were seated in a dimly lit, quiet room, with a viewing distance of approximately 60 cm from the computer screen. Stimuli were presented on a 15-inch CRT monitor. Screen resolution was 1152 × 864 pixels and a refresh rate of 60 Hz was set. Prime stimuli consisted of the digits “2,” “3,” “7,” and “8.” Target stimuli were the digits “‘1,” “4,” “6,” and “9.” The same frequency of prime–target combinations was adopted as in Heinemann et al. (2009). Inducing trials are those prime–target combinations that are frequent in only one of the two (low- or high-conflict) contexts. Test trials are prime–target combinations that are equally frequent in the two contexts (see also **Table 1**). By means of distinguishing between both trial types, the episodic learning interpretation of the CSPC effect could be put to the test. Pre- and post-masks were an array of four hash (“#”) symbols. Prime, target, and mask stimuli were centrally presented in Arial font 20 on top of the context figure. This figure was a centrally presented square of 8 cm × 8 cm that was presented in either the color cyan (RGB = 0, 255, 255) or lime (RGB = 191, 255, 0), with the two colors denoting the different conflict contexts. Participants’ responses were registered by means of a cedrus response box with 4 buttons (two on each side). Participants had to rest their middle and index finger of both hands on the corresponding buttons. Stimulus presentation and response registration were done using Tscope software (Stevens et al., 2006).

**Table 1:** Distribution of prime–target combinations in the high- and low-conflict contexts.

Prime	Target	*N* trials in low-conflict context	*N* trials in high-conflict context	Inducing trial	Test trial
2	1	7	1	X	
2	4	1	1		X
2	6	1	7	X	
2	9	1	1		X
3	1	1	1		X
3	4	7	1	X	
3	6	1	1		X
3	9	1	7	X	
7	1	1	7	X	
7	4	1	1		X
7	6	7	1	X	
7	9	1	1		X
8	1	1	1		X
8	4	1	7	X	
8	6	1	1		X
8	9	7	1	X	

*Table adopted from Heinemann et al. (2009).*

### PROCEDURE

The trial procedure closely resembled the procedure of Heinemann et al. (2009); however, stimulus presentation was synchronized with the refresh rate of the monitor (16.67 ms), leading to small differences with the timing reported^1^. Every trial started with the presentation of the context figure and a fixation cross in the middle of the screen. The context figure remained on the screen until the end of the trial, the fixation cross was presented for 700 ms. For the unconscious procedure, the fixation cross was followed by a pre-mask (66.67 ms), a prime (33.33 ms), a post-mask (66.67 ms) and the target (130 ms). For the conscious procedure, the pre-mask (66.67 ms) appeared, followed by the prime (33.33 ms), a blank (50 ms), a post-mask (16.67 ms), and the target (130 ms). Participants had 1000 ms to respond to the target. Feedback was provided when participants made an error or reacted too slow (>1000 ms). The message “Fout!” (Dutch, meaning “Wrong!”) or “Te Traag!” (Dutch, meaning “Too slow!”) was presented for 700 ms. The intertrial-interval was 700 ms.

The experiment consisted of two sessions that were conducted on two consecutive days. The first session started with two training blocks of 160 trials. In a training block, only trials from the same context (low-conflict or high-conflict) were presented. The order of high-conflict and low-conflict training blocks was counterbalanced across participants. After training, participants had to perform five blocks in which trials from the high- and low-conflict context were randomly intermixed (CSPC blocks). Each of these CSPC blocks consisted of 80 trials, with 40 trials from the high-conflict context and 40 trials from the low-conflict context. Trials from the low-conflict context consisted of 80% congruent trials and 20% incongruent trials. The high-conflict context had 20% congruent and 80% incongruent trials. After these CSPC blocks, five choice blocks of 80 trials were presented in which participants were first asked to choose between the two contexts, after which a stimulus from the chosen context appeared. Thus, on every trial the two contexts (two colored squares) were presented, one on the left and the other on the right side of the screen. Participants had to indicate which context to choose by pressing a corresponding button, after which the trial procedure as described above started. These choice blocks were included to see if participants developed a preference for one of the two figures (and their associated congruency proportion), dependent on the consciousness condition. However, because this hypothesis lies beyond the scope of the present replication attempt, we will report, but not elaborate on the results from these choice blocks. Another five CSPC blocks followed the choice blocks. The second session started with two training blocks of 160 trials. Thereafter, participants had to perform six CSPC blocks of 80 trials, followed by four choice blocks of 80 trials and again six CSPC blocks of 80 trials. At the end of the second session, participants had to complete a prime visibility task. The trial procedure of this prime visibility task was exactly the same as the CSPC blocks; crucially however, participants were instructed to respond to the prime stimulus, instead of the target. They thus had to indicate whether the prime was smaller or larger than five. Two blocks of 80 trials were presented.

The assignment of context color (lime or cyan) to proportion congruency was counterbalanced across participants. For each participant, this assignment was held constant across sessions. All participants used their right hand to perform the magnitude judgment task. They had to press the right button on the response box with their right middle finger when the digit was larger than five. They had to press the left button with their right index finger when the digit was smaller than five. In the choice blocks, participants used their left hand to make a choice. They had to press the left button if they wanted to choose the square presented on the left side of the screen, and the right button when choosing the square on the right side of the screen. Half of the participants were randomly assigned to the conscious condition, the other half of the participants to the unconscious condition.

### DATA ANALYSIS

The same outlier procedure as Heinemann et al. (2009) was used. More precisely, we excluded trials with reaction times (RTs) < 200 ms and > 1000 ms (1%). Also, error trials (7%) were removed from the RT analysis. Furthermore, in accordance with Heinemann et al. (2009), the analysis was confined to the test trials only. Mean RTs and error rates were submitted to a repeated-measures ANOVA, with conflict context (high-conflict vs. low-conflict), congruency (C vs. IC) and session (first day vs. second day) as within-subject factors and consciousness (unconscious vs. conscious) as between-subject factor.

### RESULTS

#### CSPC

**FIGURE 1:**
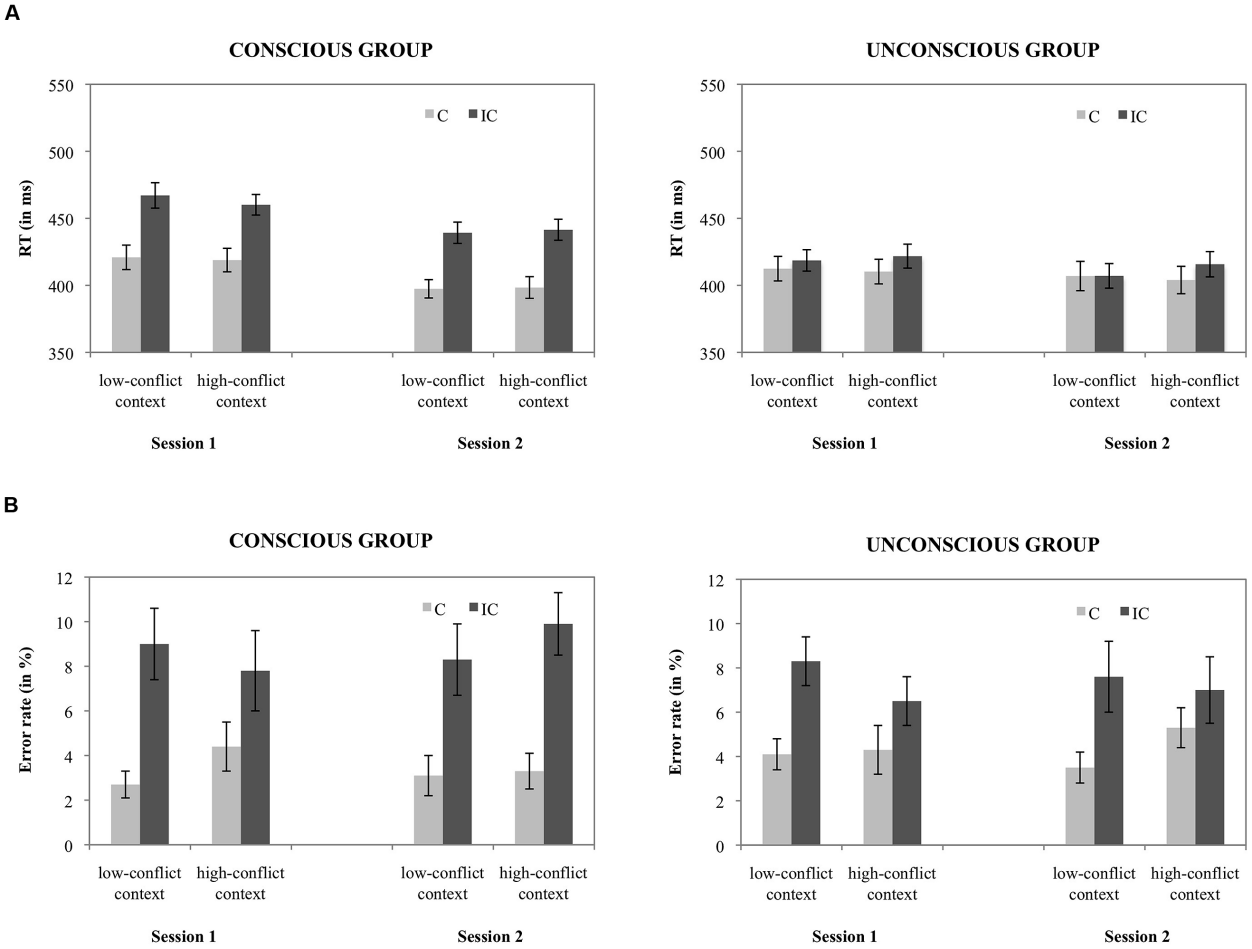
**(A)** Mean RTs for each condition, depicting the CSPC effect (calculated on the test trials only) in each session, separately for the conscious and unconscious group and **(B)** mean error rates for each condition, depicting the CSPC effect (calculated on the test trials only) in each session, separately for the conscious and unconscious group.

***RTs.*** Reaction Time results showed a main effect of session, with faster responses on the second day (*M*: 413.8 ms) than on the first day (*M*: 428.8 ms), *F*(1,38) = 16.4, *p* < 0.001, $\eta_{p}^{2}$ = 0.30. There was also a significant interaction between session and consciousness, *F*(1,38) = 4.3, *p* < 0.05, $\eta_{p}^{2}$ = 0.10, indicating that the RT difference between the first and second sessions was larger for the conscious group (*M*: 22.6 ms) than the unconscious group (*M*: 7.3 ms). Furthermore, a significant congruency effect of 25.2 ms was found, *F*(1,38) = 88.7, *p* < 0.001, $\eta_{p}^{2}$ = 0.70, as well as a significant interaction between congruency and consciousness condition, *F*(1,38) = 44.4, *p* < 0.001, $\eta_{p}^{2}$ = 0.54, with participants in the unconscious condition showing a smaller [but still significant, *F*(1,19) = 5.0, *p* < 0.05, $\eta_{p}^{2}$ = 0.21] congruency effect (*M*: 7.4 ms) compared to participants in the conscious condition (*M*: 43.1 ms). All other main and interaction effects were not significant, all *F*’s ≤ 1.9, all *p*’s ≥ 0.18. **Figure 1A** displays condition-dependent mean RTs for both consciousness groups separately.

***Error rates.*** The error rate analysis revealed a significant congruency effect, *F*(1,38) = 39.3, *p* < 0.001, $\eta_{p}^{2}$ = 0.51, indicating more errors on incongruent trials (*M*: 8.0%) than on congruent trials (*M*: 3.8%). There was also a marginally significant interaction between congruency and consciousness, *F*(1,38) = 3.0, *p* = 0.092, $\eta_{p}^{2}$ = 0.07, indicating a slightly larger congruency effect in the conscious group (*M*: 5.3%), compared to the unconscious group (*M*: 3.0%). Moreover, a significant interaction between congruency and conflict context (i.e., CSPC effect) was found, *F*(1,38) = 5.4, *p* < .005, $\eta_{p}^{2}$ = 0.13, indicating a larger congruency effect in the low-conflict context (*M*: 4.9%) than in the high-conflict context (*M*: 3.5%). All other main and interaction effects were not significant, all *F*s = 2.0, *p* ≥ 0.17. **Figure 1B** depicts mean error rates for each within-subjects condition, for both consciousness groups separately.

#### Follow-up analyses

When analyzing the data from the test trials only, we could not replicate the results of Heinemann et al. (2009). To follow-up, we also reanalyzed the data with both inducing and test trials included. A repeated-measures ANOVA was carried out, with conflict context (high-conflict vs. low-conflict), congruency (C vs. IC), session (first day vs. second day) and trial type (inducing vs. test) as within-subject factrs and consciousness (unconscious vs. conscious) as between-subjects factor.

***RTs.*** The RT analysis showed a significant main effect of trial type, *F*(1,38) = 22.1, *p* < 0.001, $\eta_{p}^{2}$ = 0.37, indicating faster responses on inducing trials (*M*: 416.2 ms) than on test trials (*M*: 421.3 ms). Furthermore, a main effect of session was found, *F*(1,38) = 20.4, *p* < 0.001, $\eta_{p}^{2}$ = 0.35, showing that participants responded faster in the second (*M*: 411.3) compared to the first session (*M*: 426.1 ms). Moreover, this difference between sessions was more pronounced for the conscious group (*M*: 21.8 ms) compared to the unconscious group (*M*: 7.6 ms), *F*(1,38) = 4.7, *p* < 0.05, $\eta_{p}^{2}$ = 0.11. Results also showed a significant congruency effect (*M*: 28.4 ms), *F*(1,38) = 134.0, *p* < 0.001, $\eta_{p}^{2}$ = 0.78, and an interaction between congruency and trial type, *F*(1,38) = 8.7, *p* < 0.01, $\eta_{p}^{2}$ = 0.19. This interaction indicated a larger congruency effect on inducing trials (*M*: 31.5 ms) compared to test trials (*M*: 25.2 ms). Importantly, the congruency effect was also modulated by consciousness, *F*(1,38) = 60.9, *p* < 0.001, $\eta_{p}^{2}$ = 0.62, with participants in the conscious group showing a larger congruency effect than the participants in the unconscious group (*M:* 47.5 ms vs. *M:* 9.3 ms). Finally a marginally significant interaction between conflict context and consciousness was found, *F*(1,38) = 3.6, *p* = 0.06, $\eta_{p}^{2}$ = 0.08. Participants in the conscious group showed faster responses in the high-conflict context (*M*: 426.3 ms) than in the low-conflict context (*M*: 427.9 ms). This was reversed for participants in the unconscious group (*M:* 411.0 ms vs. *M:* 409.6 ms). All other main and interaction effects were not significant, all *F*’s = 2.8, *p*’s ≥ 0.11.

***Error rates.*** Results from the error rate analysis showed a significant congruency effect, *F*(1,38) = 38.4, *p* < 0.001, $\eta_{p}^{2}$ = 0.50, which was furthermore modulated by consciousness, *F*(1,38) = 7.5, *p* < 0.05, $\eta_{p}^{2}$ = 0.16. Specifically, the unconscious group displayed a smaller congruency effect (*M*: 2.2%), than the conscious group (*M*: 5.5%). Furthermore, an interaction between session and conflict context was found, *F*(1,38) = 7.6, *p* < 0.01, $\eta_{p}^{2}$ = 0.17, indicating that more errors were made in the low-conflict context (*M*: 6.3%) compared to the high-conflict context (*M*: 5.8%) in session 1. However, in session 2 this difference between conflict contexts was reversed, with more errors in the high-conflict context (*M*: 6.1%) compared to the low-conflict context (*M*: 5.3%).

Results furthermore showed a three-way interaction between trial type, congruency and consciousness, *F*(1,38) = 5.0, *p* < 0.05, $\eta_{p}^{2}$ = 0.12, indicating that the congruency effect was larger on test trials (*M*: 3.0%) compared to inducing trials (*M*: 1.3%) for the unconscious group, *F*(1,19) = 6.1, *p* < 0.05, $\eta_{p}^{2}$ = 0.2. In contrast, this difference in congruency effect between trial types was negligible for the conscious group, *F* < 1.0 (*M*_test_: 5.3%; *M*_inducing_: 5.7%). Also, an interaction among trial type, conflict context, and congruency was found, *F*(1,38) = 4.3, *p* < .05, $\eta_{p}^{2}$ = .10. This three-way interaction indicated that on test trials there was a significant CSPC effect (*M*: 1.4%), *F*(1,38) = 5.4, *p* < 0.05, $\eta_{p}^{2}$ = 0.1. On inducing trials, this CSPC effect however vanished, *F* < 1.0 (*M*: –0.2%). Furthermore, results also showed an interaction among trial type, conflict context, and consciousness, *F*(1,38) = 4.7, *p* < 0.05, $\eta_{p}^{2}$ = 0.11. For the conscious group, there were more errors in the low-conflict context (*M*: 6.2%) compared to the high-conflict context (*M*: 5.5%) on inducing trials. On test trials, this difference between contexts was reversed (*M*_low-conflict_: 5.8%; *M*_high-conflict_: 6.4%). For the unconscious group, the difference between contexts on test trials was negligible (*M*_low-conflict_: 5.9%; *M*_high-conflict_: 5.8%). On inducing trials however, there were more errors in the high-conflict context (*M*: 6.2%) compared to the low-conflict context (*M:* 5.3%).

Finally, two marginally significant interactions were found. Namely, results showed a marginally significant interaction among trial type, session, and congruency, *F*(1,38) = 3.3, *p* = 0.08, $\eta_{p}^{2}$ = 0.08, indicating that the congruency effect was smaller on inducing trials (*M*: 2.7%) compared to test trials (*M*: 4.2%) in the second session, while in the first session this difference was negligible (*M*_test_: 4.0%; *M*_inducing_: 4.4%). Furthermore, a marginally significant four-way interaction among conflict context, congruency, session, and consciousness was found, *F*(1,38) = 3.8, *p* = 0.06, $\eta_{p}^{2}$ = 0.09. This interaction indicated that for the conscious group, the CSPC effect was larger in the first session (*M*: 2.9%), compared to the second session (*M*: –1.4%). For the unconscious group, there was no difference in CSPC effect between sessions (*M*_session 1_: 2.0%; *M*_session 2_: 2.4%). All other main and interaction effects were not significant, all *F*’s í 2.8, *p*’s ≥ 0.10.

#### Prime visibility

The signal detection measure (*d′*) was calculated for the conscious and unconscious group. Trials with primes smaller than 5 were considered signal, trials with primes larger than 5 were considered noise. Only trials with RTs ranging from 200 to 1000 ms were included. Mean prime visibility was 2.4 (SD: 1.6) for the conscious group, which differed significantly from 0, *t*(19) = 6.7, *p* < 0.001, *d* = 1.49. For the unconscious group, mean prime visibility was 0.4 (SD: 0.5), also deviating significantly from 0, *t*(19) = 3.6, *p* < 0.01, *d* = 0.81. Importantly, prime visibility was smaller in the unconscious group compared to the conscious group, *t*(23.2) = 5.2, *p* < 0.001, *d* = 1.68.

In a next step, we tested for the unconscious group whether there is a congruency effect when prime visibility (*d′*) is 0 (Draine and Greenwald, 1998). Therefore, we conducted a regression analysis, in which we regressed participants’ congruency effects onto the *d′* values. Congruency effects were calculated with the following formula (cfr. Heinemann et al., 2009): 100^*^ (mean RT incongruent–mean RT congruent)/mean RT congruent. The results showed a non-significant intercept, *t*(19) = 1.5, *p* = 0.16, *R*^2^ = 0.09, suggesting that with no prime visibility (*d′* = 0), there is no evidence for a congruency effect. Moreover, the slope was not significant, *t*(19) = 1.3, *p* = 0.20, *R*^2^ = 0.09, indicating that there was no correlation between prime visibility and the congruency effect.

#### Choice results

Participants in the conscious group chose the low-conflict context in 63.1% of the cases (SD: 22.2%). This mean low-conflict choice rate differed significantly from chance, *t*(19) = 2.6, *p* < 0.05, *d* = 0.59, and furthermore did not differ across the two sessions, *t* < 1. For the unconscious group, mean choice rate for the low-conflict context was 58.1% (SD: 19.7%), which differed marginally significant from chance, *t*(19) = 1.8, *p* = 0.08, *d* = 0.41. Again, no effect of session on low-conflict choice rates was found, *t* < 1. Importantly, the two groups did not differ significantly in mean choice rate for the low-conflict context, *t* < 1.

## EXPERIMENT 2

We could not replicate Heinemann et al.’s (2009) findings, in the sense that we did not find any evidence for a modulation of the size of the CSPC effect by consciousness, neither when considering only the test trials nor when considering all trials. Moreover, for the conscious condition we could not even demonstrate a CSPC effect in RTs, thereby failing to demonstrate the presence of the effect under investigation, and thereby failing to replicate the results of the original studies of Crump et al. (2006) and Crump and Milliken (2009). Importantly however, it should be noted that our experiment showed several discrepancis with Heinemann et al.’s (2009) study, thereby raising some concerns that should be addressed in a follow-up experiment before we can support the claim that context-specific control does (or does not) require consciousness.

First, the percentage of errors in the study of Heinemann et al. (2009; 32.9%) was substantially higher than the percentage obtained in our study. This discrepancy may have been caused by the difference in response deadline used. In the study of Heinemann et al. (2009) a very stringent response deadline (250 ms after target offset) was imposed, while our response deadline was set to 1000 ms. In the follow-up experiment, we opted for the same deadline procedure as Heinemann et al. (2009).

Moreover, in our experiment choice blocks were included in both sessions in which participants had to choose between the two contexts before proceeding to the magnitude judgment task. These choice blocks were included for reasons discussed elsewhere (Schouppe et al., 2014) and do not form the focus of attention in this replication study. However, one can argue that the inclusion of these choice blocks did interfere with our CSPC measure. Although follow-up analyses in which we compared the CSPC effect before and after the choice blocks do not suggest this to be the case, in Experiment 2 we strictly followed the procedure outlined in Heinemann et al. (2009) and thus removed the choice blocks.

Finally, the results of the prime visibility task of Experiment 1 suggested that the primes were still perceived at some level of consciousness (*d′* was not equal to zero). It is noteworthy however, that also in the studies of Heinemann et al. (2009) the primes were not fully invisible in the unconscious condition (i.e., *d′* = 0.79, differing significantly from zero, *t*(15) = 6.43, *p* < 0.001). We however have to acknowledge that our masking procedure was not optimal, since it did not fully prevent prime visibility. We therefore opted for a more stringent masking procedure in Experiment 2, with a prime duration of 26.67 ms and a post-mask of 13.33 ms.

## METHOD

### PARTICIPANTS

Sixty-six students^2^ (*M*: 19.4 years of age, SD: 3.6; 60 female) from Ghent University participated in the study in return for course credits. All participants were right-handed and had normal or corrected-to-normal vision. They provided written informed consent.

### STIMULI AND MATERIAL

A 19-inch CRT monitor was used with a refresh rate set to 75 Hz. Prime stimuli consisted of the digits “2,” “3,” “7,” and “8.” Target stimuli were the digits “1,” “4,” “6,” and “9.” The same frequency of prime–target combinations was adopted as Experiment 1 (see also **Table 1**). Pre- and post-masks were arrays of three symbols, randomly chosen without replacement from the following set of symbols: “%.” “?,” “&,” “§,” “β,” and “#.” Prime, target, and mask stimuli were centrally presented in Arial font 20 on top of the context figure. This figure was a centrally presented rectangle of 9 cm (*w*) × 7 cm (*h*) that was presented in either the color cyan (RGB = 0, 255, 255) or lime (RGB = 0, 255, 0), with the two colors denoting the different conflict contexts. Participants’ responses were registered using a standard keyboard. Participants were asked to rest their left and right index finger on the “F” and “J” keys respectively. Stimulus presentation and response registration were done using E-Prime (Schneider et al., 2002).

### PROCEDURE

Experiment 2 was set up as an exact replication of Heinemann et al. (2009). The trial procedure was therefore identical, besides very small differences in timing^1^. Every trial started with the presentation of the context figure and a fixation cross in the middle of the screen. The context figure remained on the screen until the end of the trial, the fixation cross was presented for 700 ms. For the unconscious procedure, the fixation cross was followed by a pre-mask (66.67 ms), a prime (26.67 ms), a post-mask (66.67 ms), and the target (146.67 ms). For the conscious procedure, the pre-mask (66.67 ms) appeared, followed by the prime (26.67 ms), a blank (53.33 ms), a post-mask (13.33 ms), and the target (146.67 ms). Participants had 250 ms to respond after target offset. A blank screen that lasted until a response was given, or until a time window of 1000 ms was exceeded, followed the target. Feedback was provided when participants made an error or reacted too slow. The message “Fout!” (Dutch, meaning “Wrong!”) was presented for 700 ms after a erroneous response was registered. The message “Te Traag!” (Dutch, meaning “Too slow!”) was presented for 700 ms when the time window of 250 ms after target offset was exceeded. Yet, responses after this time interval, were still registered with a deadline of 1000 ms. The intertrial-interval was 700 ms.

The experiment consisted of two sessions. The second session was always conducted 3 days after the first. Both sessions started with two training blocks of 160 trials (one of each conflict context). The first session consisted of five CSPC blocks of 80 trials. The second session consisted of six CSPC blocks of 80 trials and was furthermore concluded with two prime visibility blocks of 64 trials. In the training blocks, only trials from the same context (low-conflict or high-conflict) were presented. The order of high-conflict and low-conflict training blocks was counterbalanced across participants. Each CSPC block contained 40 trials from the high-conflict context and 40 trials from the low-conflict context. Trials from the low-conflict context consisted of 80% congruent trials and 20% incongruent trials. The high-conflict context contained 20% congruent and 80% incongruent trials.

The assignment of context color (lime or cyan) to proportion congruency was counterbalanced across participants. For each participant, this assignment was held constant across sessions. Participants had to press the “J” key when the digit was larger than five, and the “F” key when the digit was smaller than five. Half of the participants were randomly assigned to the conscious condition, the other half of the participants to the unconscious condition.

### DATA ANALYSIS

For the analysis of the CSPC effect, outlier trials < 200 ms and > 1000 ms were excluded (18% of all trials; 10% errors and 8% correct trials). For the RT analysis erroneous responses (38% of all trials) were also removed. Mean RTs and error rates were submitted to a repeated-measures ANOVA, with conflict context (high-conflict vs. low-conflict), congruency (C vs. IC) and session (first day vs. second day) as within-subject factors and consciousness (unconscious vs. conscious) as a between-subject factor. We first specifically focused on the test trials, however, a follow-up analysis with trial type (inducing vs. test) as an extra within-subjects factor was also carried out. Moreover, Heinemann et al. (2009) reported an RT analysis that included correct trials after the response deadline, stating that “the data pattern did not change substantially when excluding these responses” (p. 969). To check whether this was also the case in our study, we additionally analyzed the data including only trials with response times between 200 and 380 ms (68% of all trials; 27% errors and 41% correct trials).

### RESULTS

#### CSPC

**FIGURE 2:**
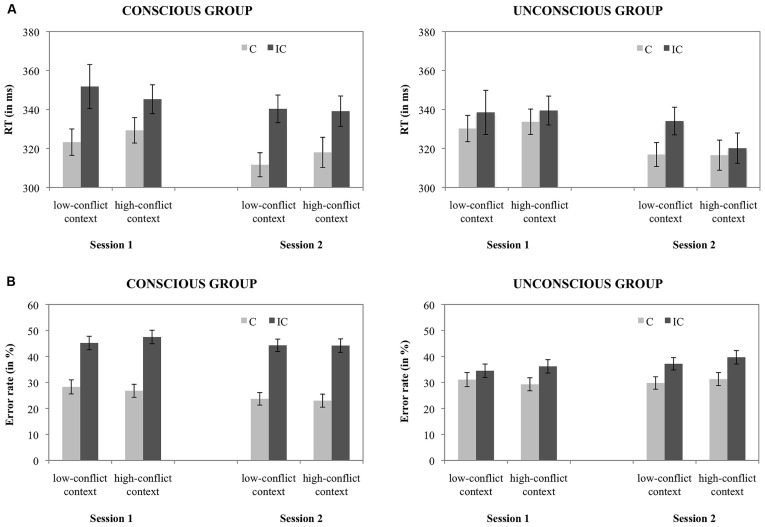
**(A)** Mean RTs for each condition, depicting the CSPC effect (calculated on the test trials only) in each session, separately for the conscious and unconscious group and **(B)** mean error rates for each condition, depicting the CSPC effect (calculated on the test trials only) in each session, separately for the conscious and unconscious group.

***RTs correct test trials with RTs > 200 ms.*** **Figure 2A** displays the mean RTs for each condition. The analysis showed a main effect of session, *F*(1,64) = 7.9, *p* < 0.01, $\eta_{p}^{2}$ = 0.11, indicating slower RTs in the first (*M*: 336.4 ms) compared to the second session (*M*: 324.6 ms). Also, a significant congruency effect of 16.1 ms was found, *F*(1,64) = 20.9, *p* < 0.001, $\eta_{p}^{2}$ = 0.25, which was more pronounced in the conscious (*M*: 23.6 ms) compared to the unconscious group (*M*: 8.7 ms), *F*(1,64) = 4.4, *p* < 0.05, $\eta_{p}^{2}$ = 0.07. Importantly, a significant conflict context × congruency interaction was found, *F*(1,64) = 4.5, *p* < 0.05, $\eta_{p}^{2}$ = 0.07, reflecting a larger congruency effect in the low-conflict context (*M*: 20.7 ms) compared to the high-conflict context (*M*: 11.6 ms). This overall CSPC effect was not modulated by consciousness, *F* < 1.0. All other main and interaction effects were not significant, all *F*’s = 1.1, all *p*’s ≥ .29. Extra analyses conducted separately for the two consciousness groups showed that the CSPC effect was significant in the unconscious group (*M*: 8.0 ms), *F*(1,32) = 4.5, *p* < 0.05, $\eta_{p}^{2}$ = 0.12, but not in the conscious group (*M*: 10.0 ms), *F*(1,32) = 1.7, *p* > 0.1, $\eta_{p}^{2}$ = 0.05.

***RTs correct test trials with RTs > 200 ms and = 380 ms.*** However, the result pattern was different when only considering correct trials between 200 and 380 ms. The 2 (session) × 2 (conflict context) × 2 (congruency) × 2 (consciousness) repeated-measures ANOVA then revealed a marginally significant main effect of conflict context, *F*(1,64) = 3.8, *p* = 0.06, $\eta_{p}^{2}$ = 0.06, with larger RTs for the high-conflict (*M*: 294.4 ms) compared to the low-conflict context (*M*: 291.2 ms). Moreover, this effect of conflict context was modulated by consciousness, *F*(1,64) = 6.0, *p* < 0.05, $\eta_{p}^{2}$ = 0.09, with participants in the conscious group showing a larger effect of conflict context (*M*: 7.1 ms), than participants in the unconscious group (*M*: –0.8 ms). Furthermore, a marginally significant interaction between conflict context and session was found, *F*(1,64) = 3.4, *p* = 0.07, $\eta_{p}^{2}$ = 0.05, as well as a marginally significant interaction among conflict context, congruency and session, *F*(1,64) = 3.4, *p* = 0.07, $\eta_{p}^{2}$ = 0.05. This three-way interaction reflects a reversed CSPC effect of –6.9 ms in the first session, and a normal CSPC effect of 7.4 ms in the second session. All other main and interaction effects were not significant, all *F*’s = 2.5, all *p*’s ≥ 0.12. Extra analyses for the conscious and unconscious group separately showed neither a significant CSPC effect nor an interaction between the CSPC effect and session, for either group, all *F*’s = 2.1, all *p*’s ≥ 0.16.

***Error rates test trials with RTs > 200.*** **Figure 2B** displays the mean error rates for each condition of interest. The error rate analysis showed a significant congruency effect of 13.2%, *F*(1,64) = 71.0, *p* < 0.001, $\eta_{p}^{2}$ = 0.53, which was furthermore influenced by consciousness, *F*(1,64) = 18.1, *p* < .001, $\eta_{p}^{2}$ = 0.22. As in RTs, the unconscious group displayed a smaller congruency effect than the conscious group (*M*: 6.5% vs. *M:* 19.9%). Furthermore, an interaction between session and consciousness was found, *F*(1,64) = 5.7, *p* < 0.05, $\eta_{p}^{2}$ = 0.08, indicating that participants in the conscious group made less errors in the second (*M*: 33.8%) compared to the first session (*M*: 37.0%). In the unconscious group this pattern however reversed, with more errors in the second (*M*: 34.5%) compared the first session (*M*: 32.8%). All other main and interaction effects were not significant, all *F*’s = 1.8, all *p*’s ≥ 0.18. Extra analyses on the two consciousness groups separately showed no sign of an interaction between conflict context and congruency, or of an interaction between session, conflict context and congruency, all *F*’s < 1.

***Error rates test trials with RTs > 200 ms and = 380 ms.*** In a next step, we also analyzed the accuracy scores of trials with RTs > 200 ms and = 380 ms and found similar results.

#### Follow-up analyses

As in Experiment 1, we also conducted follow-up analyses where a repeated-measures ANOVA was carried out on RTs and error rates, with conflict context (high-conflict vs. low-conflict), congruency (C vs. IC), session (first day vs. second day) and trial type (inducing vs. test) as within-subject factors and consciousness (unconscious vs. conscious) as between-subjects factor.

***RTs correct trials with RT > 200 ms.*** Results again showed a main effect of session, *F*(1,64) = 12.9, *p* = 0.001, $\eta_{p}^{2}$ = 0.17, indicating faster RTs in the second (*M*: 324.0 ms) compared to the first session (*M*: 335.7 ms). Also, a main effect of congruency was found, *F*(1,64) = 52.5, *p* < 0.001, $\eta_{p}^{2}$ = 0.45, with congruent trials (*M*: 322.0 ms) responded to faster than incongruent trials (*M*: 337.7 ms). This congruency effect was larger in the conscious group (*M*: 21.9 ms), compared to the unconscious group (*M*: 9.5 ms), *F*(1,64) = 8.2, *p* < 0.01, $\eta_{p}^{2}$ = 0.11. Furthermore, a significant interaction between trial type and consciousness was found, *F*(1,64) = 6.6, *p* < 0.05, $\eta_{p}^{2}$ = 0.09. Participants in the conscious group responded faster to inducing trials (*M*: 327.1 ms) compared to test trials (*M*: 332.3 ms). In contrast, participants in the unconscious group were faster on test trials (*M*: 328.7 ms) than on inducing trials (*M*: 331.4 ms). Also, the results showed a marginally significant interaction between session, consciousness and conflict context, *F*(1,64) = 3.1, *p* = 0.09, $\eta_{p}^{2}$ = 0.05. Finally, a three-way interaction among trial type, conflict context, and congruency, *F*(1,64) = 4.0, *p* = 0.05, $\eta_{p}^{2}$ = 0.06, was found, indicating a larger CSPC effect for test trials (*M*: 9.0 ms), compared to the inducing trials (*M*: –2.9 ms). All other main and interaction effects were not significant, all *F*’s = 2.2, all *p*’s ≥ 0.15.

***RTs correct trials with RTs > 200 ms and = 380 ms.*** A different result pattern emerged when analyzing only trials with RTs > 200 ms and = 380 ms. One participant was excluded due to a missing cell in one of the conditions. A main effect of trial type was found, *F*(1,63) = 4.2, *p* < 0.05, $\eta_{p}^{2}$ = 0.06, indicating slightly faster responses on test trials (*M*: 293.2 ms) compared to inducing trials (*M*: 295.2 ms). This effect of trial type interacted with conflict context, *F*(1,63) = 9.3, *p* < 0.01, $\eta_{p}^{2}$ = 0.13, in the sense that for the low-conflict context, test trials (*M*: 291.6 ms) were faster responded to than inducing trials (*M*: 296.5 ms). For the high-conflict context, this effect numerically reversed (*M*_test_: 294.9 ms vs. *M*_inducing_: 293.9 ms). The effect of trial type × conflict context was moreover more pronounced in the conscious compared to the unconscious group, and in the first compared to the second session, as indicated by the significant three-way interactions among trial type, conflict context, and consciousness on the one hand, *F*(1,63) = 5.9, *p* < 0.05, $\eta_{p}^{2}$ = 0.09, and among trial type, conflict context, and session on the other hand, *F*(1,63) = 5.1, *p* < 0.05, $\eta_{p}^{2}$ = 0.08. Furthermore, a significant four-way interaction among session, trial type, conflict context, and congruency was shown, *F*(1,63) = 6.6, *p* < 0.05, $\eta_{p}^{2}$ = 0.09. This interaction indicated that for the test trials, the CSPC effect evolved from –3.8 ms to 8.6 ms over the two sessions. For the inducing trials this trend was reversed, with a CSPC effect of 8.6 ms in session 1 and a CSPC effect of 0.1 ms in session 2. Finally, the results showed three marginally significant interactions. An interaction between session and consciousness was found, *F*(1,63) = 3.7, *p* = 0.06, $\eta_{p}^{2}$ = 0.06, showing that for the conscious group there was no difference in RTs between both sessions (*M*_first session_: 291.1 ms vs. *M*_second session_*:* 291.7 ms). For the unconscious group, participants responded slower in the first (*M*: 300.6 ms) compared to the second session (*M*: 293.5 ms). Also, the results showed a marginally significant interaction between trial type and congruency, *F*(1,63) = 3.4, *p* = 0.07, $\eta_{p}^{2}$ = 0.05, indicating a smaller congruency effect on test trials (*M*: 0.0 ms) compared to inducing trials (*M*: 3.5 ms). Finally, a marginally significant interaction between conflict context and congruency, *F*(1,63) = 2.8, *p* = 0.10, $\eta_{p}^{2}$ = 0.04, denoted a (small) CSPC effect of 3.0 ms. All other main and interaction effects were not significant, all *F*’s = 2.6, all *p*’s ≥ 0.11.

***Errors rates trials with RTs > 200 ms.*** More errors were made on incongruent (*M*: 41.6%) compared to congruent trials (*M*: 28.3%), *F*(1,64) = 130.1, *p* < 0.001, $\eta_{p}^{2}$ = 0.67. This congruency effect was modulated by consciousness, *F*(1,64) = 37.1, *p* < 0.001, $\eta_{p}^{2}$ = 0.37, with a larger congruency effect in the conscious (*M*: 20.5%) compared to the unconscious group (*M*: 6.2%). Also, the congruency effect was larger in the second (*M*: 14.8%) compared to the first session (*M*: 12.0%), *F*(1,64) = 4.7, *p* < 0.05, $\eta_{p}^{2}$ = 0.07. Furthermore, an interaction between session and consciousness was found, *F*(1,64) = 8.2, *p* < 0.01, $\eta_{p}^{2}$ = 0.11. The conscious group displayed more errors in the first (*M*: 37.4%) compared to the second session (*M*: 34.5%). This effect was reversed for the unconscious group (*M*_first session_: 32.8% vs. *M*_second session_: 35.1%). All other main and interaction effects were not significant, all *F*’s = 2.7, all *p*’s ≥ 0.10.

***Error rates trials with RTs > 200 ms and = 380 ms.*** The results of this additional analysis were highly similar. We found a significant main effect of congruency, *F*(1,64) = 165.5, *p* < 0.001, $\eta_{p}^{2}$ = 0.72, and two significant interaction effects: congruency × consciousness, *F*(1,64) = 43.2, *p* < 0.001, $\eta_{p}^{2}$ = 0.4, and session × consciousness, *F*(1,64) = 10.9, *p* < 0.01, $\eta_{p}^{2}$ = 0.15. A marginally significant main effect of session was also found, *F*(1,64) = 3.5, *p* = 0.065, $\eta_{p}^{2}$ = 0.05, as well as a marginally significant main effect of consciousness, *F*(1,64) = 3.0, *p* = 0.09, $\eta_{p}^{2}$ = 0.04. All other main and interaction effects were not significant, all *F*’s = 2.4, all *p*’s ≥ 0.12.

#### Prime visibility

Analogously to the analysis of the CSPC effect, only trials with RTs > 200 ms and < 1000 ms were considered for the calculation of the signal detection measure. For the conscious group, the mean level of prime visibility (*d′* value) was 0.34 (SD: 0.45), which deviated significantly from 0, *t*(32) = 4.3, *p* < 0.001, *d* = 0.76. In contrast, for the unconscious group the mean *d′* value was 0.10 (SD: 0.45), which did not differ significantly from 0, *t*(32) = 1.2, *p* > 0.1, *d* = .21. Importantly, the *t*-test comparing both groups in terms of prime visibility was significant, *t*(64) = 2.2, *p* < 0.05, *d* = 0.56.

Furthermore, we conducted a regression analysis, in which we regressed each participants’ congruency effect of the unconscious group (calculated as 100^*^ [mean RT incongruent-mean RT congruent]/mean RT congruent) onto the *d’* value. The results showed a non-significant intercept, *t*(31) = 1.3, *p* > 0.10, *R*^2^ = 0.03 suggesting that with no prime visibility (*d′* = 0), there is no evidence for a congruency effect. Moreover, the slope was not significant, *t*(31) = 1, *p* > 0.10, *R*^2^ = 0.03, indicating that there was no correlation between prime visibility and the congruency effect.

## GENERAL DISCUSSION

The literature on consciousness and control is expanding, yet the findings remain mixed and do not allow a comprehensive conclusion. Some studies showed that cognitive control can act subconsciously, demonstrating conflict-driven adaptations to subliminally presented conflict stimuli (Van Gaal et al., 2010a; Desender et al., 2013; Reuss et al., 2014). Others argued the opposite, showing an absence of typical cognitive control effects under subconscious conditions (Kunde, 2003; Frings and Wentura, 2008; Heinemann et al., 2009; Ansorge et al., 2011). These mixed findings can partly be explained in terms of different control operations requiring different levels of awareness. Yet, before coming to such a conclusion, replication studies are highly necessary. Therefore, the aim of this study was to replicate Heinemann et al.’s (2009) experiment, which was the first to control for episodic learning confounds (Hommel et al., 2004; Schmidt, 2013) when studying the role of consciousness in cognitive control.

Heinemann et al. (2009) used a masked number priming task where two contexts denoted the relative frequency of incongruent prime–target combinations. They showed that the CSPC effect could be observed in the conscious group, but not in the unconscious group, suggesting that cognitive control only acts consciously. However, in two experiments with high statistical power, we failed to reproduce these results. In Experiment 1, we found evidence for a CSPC effect – albeit only in error rates – that was not modulated by consciousness. Experiment 2, set up as an exact replication of Heinemann et al. (2009) showed an overall CSPC effect – now only in RTs – that again did not interact with consciousness. If anything, our results indicate that the CSPC effect does not depend on awareness, contrary to the conclusion of Heinemann et al. (2009). This is in line with a recent study by Reuss et al. (2014) where the visibility of primes was manipulated in several masked priming experiments, while the conflict context was determined by the format of either the prime or the target. By associating the context with either prime or target, they could furthermore investigate whether the temporal proximity between context information and conflict is of importance for unconscious cognitive control to occur. Importantly, in Experiments 1 and 2 their results showed a CSPC effect, with visible, as well as masked primes, and when the context was set by the format of the primes, as well as by the targets. In Experiments 3 and 4, Reuss et al. (2014) used test and inducing trials to exclude the event learning account. Here, they found a CSPC effect on test trials in the unconscious condition, but only when the context was determined by the format of the prime. When the context was represented by the format of the target, a CSPC effect was observed with visible but not with masked primes.

Contrary to Reuss et al. (2014), it should be noted that in our study the dissociation between inducing and test trials did not turn out as expected (Crump and Milliken, 2009). Surprisingly, in both experiments the CSPC effect was smaller, if not absent, on the inducing trials compared to the test trials. Unfortunately, Heinemann et al. (2009) did not report the data of inducing trials, so we cannot directly compare our results. Given the lack of a CSPC effect on inducing trials however, together with the fact that we did not find a CSPC effect in RTs in Experiment 1, and the disappearance of the CSPC effect in Experiment 2 when considering only trials within the 380 ms deadline, one might question the overall reliability and robustness of the effect under investigation. In the literature, CSPC effects have always been rather small in size and some studies have reported boundary conditions for its occurrence (Crump et al., 2006, 2008), suggesting that this effect may not be ideally suited to investigate the role of consciousness on cognitive control. Alternatively, one might consider paradigms where episodic learning effects can be excluded by design (without the need for inducing trials or exclusion of trials afterwards). For instance, Duthoo et al. (submitted) designed conflict paradigms in which the congruency sequence effect could be investigated in its “pure” form, free from feature integration and contingency learning by design (see also Kim and Cho, 2014). Using such a contingency- and feature-unbiased conflict task would enable us to investigate consciousness and cognitive control, while effectively controlling for all alternative low-level learning accounts, without the need to dissociate inducing and test trials.

Finally, it is important to emphasize one critical deviation from the results of Heinemann et al. (2009). Specifically, the difference in *d′* between both conditions was much smaller in our Experiment 2 (conscious: *d′* = 0.34; unconscious: *d′* = 0.098) compared to Heinemann et al. (2009; conscious: *d′* = 2.14; unconscious: *d′* = 0.79). It is possible that this similarity in prime visibility could account for the similar CSPC results in both consciousness conditions in Experiment 2. One could even argue that the magnitude of the *d′* measure of the conscious condition indicates unconscious – rather than conscious – processing of the prime, since it appears to be heavily masked. Yet, the *d′* measure differed significantly from zero in the conscious group, while it did not deviate from zero in the unconscious group. Also, both consciousness conditions differed significantly in terms of *d′*. These results strongly support the conclusion that primes were consciously perceived in the conscious group and that prime visibility is not similar across both conditions. Moreover, the results of Experiment 1 further contradict the notion that similarity in prime visibility accounts for the similar CSPC results. Here, the prime visibility measures (conscious: *d′* = 2.4, unconscious: *d′* = 0.4) were more similar to Heinemann et al. (2009), and we still found a CSPC effect in both conditions.

In sum, we failed to replicate the results described by Heinemann et al. (2009). With some caution, our data seem to suggest that context-specific control can operate outside the border of awareness as we observed a CSPC effect on frequency-unbiased test trials in both the conscious and unconscious group. However, the CSPC effect was only observable on test trials, and not on inducing trials. We moreover failed to find a CSPC effect on RTs in Experiment 1, and also in Experiment 2 the effect was not observed consistently, as the results did not show a CSPC effect when only considering trials that were responded to within the response deadline. Based on these observations, we believe caution is warranted when using the CSPC effect as a marker of cognitive control.

## Conflict of Interest Statement

The authors declare that the research was conducted in the absence of any commercial or financial relationships that could be construed as a potential conflict of interest.

## Footnotes

1 Heinemann et al. (2009) did not report which refresh rate of the monitor they used. Please note however that the timing as reported in Heinemann et al. (2009) is unrealistic because to our knowledge one cannot obtain a timing of 70 ms (pre-mask), 26 ms (prime), 60 ms (blank), and 10 ms (post-mask), when stimulus presentation is synchronized with the refresh rate of a monitor. In Experiment 1, we unfortunately did not register the precise timing of the presentation of the target, so that we can only report an approximate value for this presentation time.

2 Based on the original dataset of Heinemann et al. (2009), we calculated the number of participants needed to achieve 95% power (given α = 0.05) for the between-subject comparison of the CSPC effect (conscious vs. unconscious). More specifically, in Heinemann’s study, the CSPC effect differed significantly between the conscious and unconscious group, *t*(27) = 2.5, *p* < 0.05, *d* = 0.91. Based on this Cohen’s *d* effect size, we calculated using G-Power 3 (Faul et al., 2007) that 36 participants had to be tested to achieve 95% power.
